# RADB: a database of rheumatoid arthritis-related polymorphisms

**DOI:** 10.1093/database/bau090

**Published:** 2014-09-15

**Authors:** Ruijie Zhang, Meiwei Luan, Zhenwei Shang, Lian Duan, Guoping Tang, Miao Shi, Wenhua Lv, Hongjie Zhu, Jin Li, Hongchao Lv, Mingming Zhang, Guiyou Liu, He Chen, Yongshuai Jiang

**Affiliations:** ^1^College of Bioinformatics Science and Technology, Harbin Medical University, Harbin 150086, China, ^2^Yiwu Hospital, Zhejiang University, Yiwu 322000, China, ^3^Genome Analysis Laboratory, Tianjin Institute of Industrial Biotechnology, Chinese Academy of Sciences, Tianjin, 300308, China, ^4^Depatment of Pathology, Harbin Medical University, Harbin 150086, China

## Abstract

Rheumatoid arthritis (RA) is an autoimmune disease that has a complex genetic basis. Therefore, it is important to explore the genetic background of RA. The extensive recent application of polymorphic genetic markers, especially single nucleotide polymorphisms, has presented us with a large quantity of genetic data. In this study, we developed the Database of Rheumatoid Arthritis-related Polymorphisms (RADB), to integrate all the RA-related genetic polymorphisms and provide a useful resource for researchers. We manually extracted the RA-related polymorphisms from 686 published reports, including RA susceptibility loci, polymorphisms associated with particular clinical features of RA, polymorphisms associated with drug response in RA and polymorphisms associated with a higher risk of cardiovascular disease in RA. Currently, RADB V1.0 contains 3235 polymorphisms that are associated with 636 genes and refer to 68 countries. The detailed information extracted from the literature includes basic information about the articles (e.g. PubMed ID, title and abstract), population information (e.g. country, geographic area and sample size) and polymorphism information (e.g. polymorphism name, gene, genotype, odds ratio and 95% confidence interval, *P*-value and risk allele). Meanwhile, useful annotations, such as hyperlinks to dbSNP, GenBank, UCSC, Gene Ontology and Kyoto Encyclopedia of Genes and Genomes pathway, are included. In addition, a tool for meta-analysis was developed to summarize the results of multiple studies. The database is freely available at http://www.bioapp.org/RADB.

**Database URL:** http://www.bioapp.org/RADB.

## Introduction

Rheumatoid arthritis (RA) is a systemic inflammatory autoimmune disorder affected by genetic and environmental factors (1). The genetic component of RA has been estimated to be between 50 and 60% (2). Unlike single-gene disorders, RA is believed to be associated with multiple genes and their interactions (3). The strongest association has been shown to be with the *HLA-DRB1* region (6p21), explaining ∼30% of the total genetic effect (4). In addition to the HLA region, non-HLA genes (e.g. *PTPN22*, *PADI4*) have also been reported to contribute to RA susceptibility (5, 6). Currently, many loci that have convincing evidence for association with RA have been identified. However, the results are often poorly replicated, especially in different populations, increasing the complexity of the research. Collecting and collating the information about RA risk loci will facilitate systematic exploration of the genetic mechanisms of RA. Currently, there are several genetic association databases [e.g. Online Mendelian Inheritance in Man (OMIM) (7) and the Genetic Association Database (GAD) (8)] to store disease susceptibility loci. OMIM focuses on high-quality data of high significance for Mendelian disorders. Although in recent years, non-Mendelian diseases (also known as ‘common’ or ‘complex’ diseases) have been included, some biases still exist because of its history. In addition, OMIM is largely based on text and is a narrative history of disease research; thus, it is not designed to compare or analyze large sets of genetic data. More importantly, association studies of non-Mendelian diseases often have low-significance values, and findings of lower significance or negative findings are not routinely included in OMIM. Although GAD overcomes some disadvantages of OMIM, it is not a specialized database for RA, and polymorphisms associated with RA are not collected comprehensively. In addition, polymorphism genotype data are not collected in GAD, making some studies (e.g. meta-analysis) difficult. Therefore, a comprehensive, exhaustive and specialized database that includes all available genetic association study data from the published literature is urgently needed.

In addition to RA susceptibility, its clinical features [e.g. rheumatoid factor (RF) status, age of onset], drug response and cardiovascular (CV) events are also significantly influenced by genetic variation. Integrated management of these genetic variations and their relevant experimental information is also necessary, but so far, there is no database in which to store them.

Here, we present the Database of Rheumatoid Arthritis-related Polymorphisms (RADB) to integrate and analyze RA-related genetic polymorphisms extracted from published papers. The information collected comprises susceptibility loci for RA, polymorphisms associated with the clinical features of RA, polymorphisms associated with drug response in RA and polymorphisms associated with a higher risk of CV disease in RA. We not only collected polymorphisms that are significantly associated with RA, but also collected polymorphisms of lower significance and non-associated polymorphisms from RA-related research. To facilitate the users’ ability to summarize the results of multiple studies, a linked tool for meta-analysis was developed. In addition, useful annotations, such as those from dbSNP (9), the National Centre for Biotechnology Information (NCBI) GenBank (10), University of California Santa Cruz (UCSC) (11) and Gene Ontology (GO) (12), were integrated into RADB to complement and extend the information from the literature.

## Data collection and database content

### Data collection

We searched the PubMed database with following keywords: ((polymorphism [Title/Abstract] OR polymorphisms [Title/Abstract] OR GWAS [Title/Abstract] OR GWA [Title/Abstract]) AND rheumatoid arthritis [Title/Abstract]) NOT review [Publication Type]. We obtained ∼2000 publications. After manually scanning the list, 686 studies were included in RADB, comprising 21 candidate gene linkage analysis studies, 640 candidate gene association studies and 25 genome-wide association studies (GWAS).We extracted the important information from these reports, including basic information about the article [e.g. PubMed ID (PMID), title and abstract], population information (e.g. country, geographic area and sample size) and polymorphism information [e.g. polymorphism name, gene, genotype, odds ratio (OR) with 95% confidence interval (CI), *P*-value and risk allele].

Different laboratories may have different standards to describe the same polymorphism or gene; it is essential to standardize them. Polymorphisms may have multiple names: for example, rs2476601, *PTPN22* 1858C/T and *PTPN22* R620W represent the same polymorphism. To standardize the name, we merged the synonyms for each polymorphism. For genes, we used the approved gene name/symbol and Entrez Gene ID.

To obtain more information, we added hyperlinks to external databases: dbSNP or the IMGT/HLA database (13) for polymorphisms; and the NCBI Gene (14), EMBL-EBI (15), sequence databases (NCBI GenBank, RefSeq (16) and Unigene (17)), protein databases (Uniprot (18), Pfam (19) and Prosite (20)) and biological pathway databases [GO and Kyoto Encyclopedia of Genes and Genomes (KEGG) pathway (21)] for genes.

### Data categories

Using our criteria, we identified 3235 polymorphisms from 636 genes. The polymorphisms were divided into four classes: (i) susceptibility loci for RA; (ii) polymorphisms associated with particular clinical features of RA; (iii) polymorphisms associated with drug response in RA; and (iv) polymorphisms associated with a higher risk of CV disease in RA. Although these four classes are not independent—for example, *PTPN22* rs2476601 exists in all four classes—we believe that such classification will enable users to interrogate our database quickly and in more depth. The primary relationships between the classes are shown in Table 1.

**Table 1.** Main relationships between the classes

Relationship	*n*
Class I ∩ Class II	299
Class I ∩ Class III	73
Class II ∩ Class III	37
Class I ∩ Class II ∩ Class III	34

Class I represents susceptibility loci, Class II represents polymorphisms associated with clinical features, Class III represents polymorphisms associated with drug response, ∩ represents intersection. The number of polymorphisms associated with a higher risk of cardiovascular events is not reflected in this table because there are insufficient reports (only 24).

#### (i) Susceptibility loci for RA

Currently, RADB contains 623 reports that examined the relationships between 2855 polymorphisms (597 genes/regions) and RA susceptibility. Among these, 562 polymorphisms (226 genes/regions) have *P* values < 0.05, 418 polymorphisms (180 genes/regions) have *P* values < 1 × 10^−^^3^ and 242 polymorphisms (113 genes/regions) have *P* values < 1 × 10^−^^5^. The strongest genetic association with RA has been found for *HLA-DRB1* alleles on chromosome 6p21. In addition to the HLA region, non-HLA gene polymorphisms, including *PTPN22* rs2476601 (5, 22), *STAT4* rs7574865 (23, 24), *TRAF1/C5* rs3761847 (25, 26), *CTLA4* rs3087243(27, 28) and *PADI4* rs2240340 (6, 29), have also been reported to be strongly associated with RA susceptibility. However, these risk alleles differ among ethnic populations. HLA-DRB1*0401, 0404 and *0101 are the most common RA risk alleles among those of European ancestry (30, 31), while HLA-DRB1*0405 is the most common RA susceptibility allele for East Asian populations (32, 33). *PTPN22* rs2476601 is a susceptibility locus for people of European ancestry, but is not associated with RA in Asian populations (6, 34, 35). Although an association has been reported between *PADI4* rs2240340 and RA in East Asian populations, it was not replicated in those of European ancestry (36, 37). It was important, therefore, for our database to contain population information. The genes and genetic regions that have the strongest association with RA susceptibility are shown in Supplementary File S1 on the Web site: http://www.bioapp.org/research/RA.

#### (ii) Polymorphisms associated with the clinical features of RA

Currently, RADB contains 46 reports that examined the relationships between 156 polymorphisms (55 genes/regions) and clinical features of RA. Among these, 19 polymorphisms (18 genes/regions) have *P* values < 0.05 and 11 polymorphisms (5 genes/regions) have *P* values < 1 × 10^−^^3^. The main clinical features analyzed include anti-citrullinated peptide antibodies (ACCP) status, RF status, age of onset and the activity/severity of RA. For example, *HLA-DRB1* SE-alleles not only affect disease susceptibility, but also influence RF status, ACCP status, age of onset and the activity/severity of RA (38–42). In non-HLA regions, *IL10* rs1800896 (–1082G/A) is associated with RF status (43). *PTPN22* rs2476601 is associated with ACCP status (38). *IL8* rs2227306 (781C/T) is associated with age of onset (44). RA activity/severity is influenced by *IL6* rs1800795 (–174G/C), *IL2* –330G/T and *TNFA* rs1800629 (–308A/G) (45–47).

#### (iii) Polymorphisms associated with drug response in RA

Disease-modifying anti-rheumatic drugs [e.g. methotrexate (MTX)] and biologics [e.g. anti-tumor necrosis factor (anti-TNF) agents] are the mainstay of treatment for RA. However, inconsistent response to these drugs is often observed, with considerable variability in both efficacy and toxicity (48). Currently, RADB contains 31 reports that examined the relationships between 176 polymorphisms (11 genes/regions) and drug response in RA. Among these, 40 polymorphisms (7 genes/regions) have *P* values < 0.05, and 13 polymorphisms (4 genes/regions) have *P* values < 1 × 10^−^^3^. For example, *RFC* G80A, *ATIC* rs4673993, *SHMT1* C1420T, *SLC19A1* rs1232027 (G80A), *HLA-DRB1*, *MTHFR* rs1801133 (677C/T) and *MTHFR* rs1801131 (1298A/C) have been found to be associated with response to MTX treatment in patients with RA (49–54). The response to anti-TNF agents has been described to be associated with *TNFA* rs1800629 (–308G/A), *FCGR3A* rs396991 (F158V), *AFF3* rs10865035 and *CD226* rs763361 (Gly307Ser) (55–59). The toxicity of MTX treatment has been shown to be associated with *RFC1* A80G, *MDR1* C3435T and *MTHFR* rs1801131 (60).

#### (iv) Polymorphisms associated with a higher risk of CV disease in RA

RA is associated with an increased risk of CV events, causing increased CV morbidity and mortality (61). Currently, RADB contains 48 reports that examined the relationships between 83 polymorphisms (37 genes/regions) and a higher risk of CV in RA. Among these, 18 polymorphisms (17 genes/regions) have *P* values < 0.05, and 2 polymorphisms (2 genes/regions) have *P* values < 1 × 10^−^^3^, namely, LCE3C_LCE3B-del and CCR5 d32 (62–64). Although the number of polymorphisms associated with CV events is still relatively small, we expect the amount of data to expand on further research.

### Meta-analysis module

The results of different association studies often show inconsistencies. A comprehensive evaluation of these results is important. Thus, we developed a module to perform a direct meta-analysis on the polymorphisms in RADB. Users can choose the parameters, such as the type of study (e.g. case–control study), the assumed risk allele and the genetic model. In addition, users can either analyze just their own data or supplement it with RADB data. In our meta-analysis module, the OR and 95% CI are calculated to assess the strength of association. Statistical heterogeneity among the studies is assessed with Woolf's test (65). A fixed-effects model using the Mantel–Haenszel method (66) and the random effects model of DerSimonian and Laird (67) are used to summarize the results. The summary results are presented in tabular form and forest plots. We also provide a funnel plot to detect publication biases. The full paper hyperlinks of the included research are offered to facilitate the inquiries of users that want more detailed information of samples.

### Querying the database

To meet the needs of different users, we offer different ways to search our database, including searching by polymorphism, searching by gene, searching by population, searching by different types of research (including candidate gene linkage analysis studies, candidate gene association studies and GWAS) and searching by chromosome.

**Figure 1.**
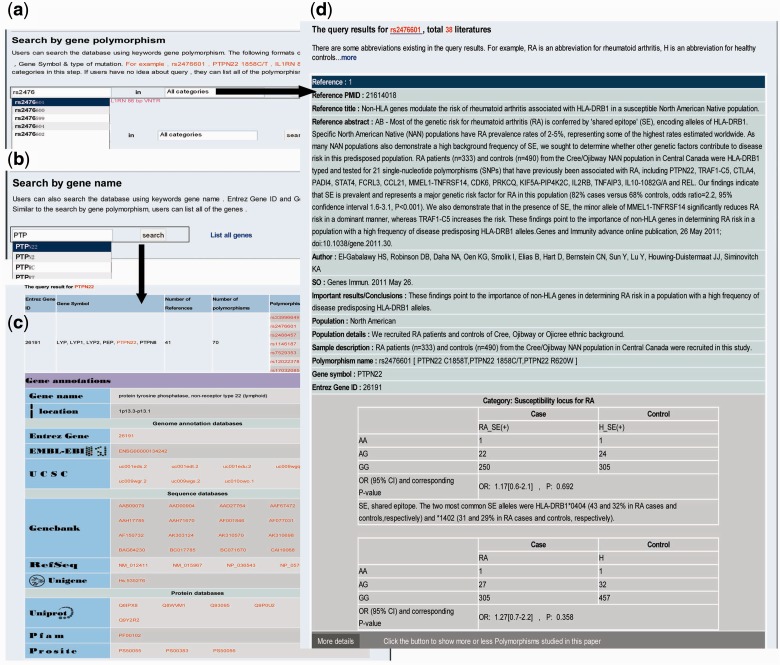
Examples of searching RADB by polymorphism name and gene name. (**a**) Searching RADB by polymorphism name. (**b**) Searching RADB by gene name. (**c**) Query results retrieved by searching with gene name. (**d**) Query results retrieved by searching with polymorphism name.

Searching RADB by polymorphism name is a basic function. There are several types of polymorphism, such as single nucleotide polymorphisms, HLA alleles and microsatellites. Users can use the dbSNP ‘rs’ number, gene symbol plus mutation position or gene symbol plus type of mutation to query RADB: for instance ‘rs2476601’, ‘*PTPN22* 1858C/T’, ‘HLA-DRB1*0401’ or *IL1RN* 86 bp VNTR’ (Figure 1a). As mentioned above, the data are divided into four classes. Users can optionally choose a category of interest at this step. To facilitate ease of use, an auto-complete function has been used. The query results are reference centered (i.e*.* each record is a reference) and are displayed by publication date on a new page (Figure 1d). For instance, if a polymorphism has been described in 10 references there will be 10 records. The query results include basic information about the articles (e.g. PMID, title, source and important results/conclusions), population information (e.g. geographic area, population, population details and sample description) and polymorphism information (e.g. polymorphism name, gene symbol, Entrez Gene ID, genotype, OR and 95% CI, *P*-value and risk allele). If an article also examined other polymorphisms, a button will appear at the bottom of each record; users can click this button to display the other polymorphisms studied in the same paper.

Users can query the database using a keyword gene name (Fig. 1b) or list all the genes in RADB. Both Entrez Gene ID and Gene Symbol are currently supported, (e.g. 26191, *PTPN22*). The results are displayed on a new page (Fig. 1c). The results include gene-related information (e.g. number of references, number of polymorphisms and polymorphism list) and hyperlinked gene annotations (e.g. gene name, location, Entrez Gene, EMBL-EBI, UCSC, GenBank, RefSeq, Unigene, Uniprot, Pfam, Prosite, GO and KEGG pathway).

In addition to querying RADB by polymorphism name and gene name, users can search RADB by population, type of research and chromosome. If the user queries RADB by population, the results will list all studies undertaken within the same population and their corresponding polymorphisms. If the user searches RADB by type of research, the results will list all reports of the same study type and their corresponding polymorphisms. If the user searches RADB by chromosome (such as ‘6’, ‘X’ or ‘mitochondrion’), the results will list all the genes and their corresponding polymorphisms located in the queried chromosome or chromosomal region.

### Submitting new data

To continually improve our database, we welcome the ongoing submission of new data. The submission process is simple. Users are only required to submit the article’s PMID and the corresponding polymorphism names. We will verify and input the data, if they meet our requirements, as soon as possible by manually filtering and sending data.

## Discussion and conclusion

Over the past 4 years, we have extracted a large number of polymorphisms associated with RA from the published literature. These polymorphisms were collected and collated manually to obtain detailed and reliable data. The polymorphisms are associated with different phenotypes in different studies. For example, the purpose of some studies is to determine whether a certain polymorphism is an RA susceptibility locus; thus, we need to examine the association between the polymorphism and the presence of RA. In these cases, the samples are patients with RA and healthy controls. However, the purpose of other studies is to determine whether a certain polymorphism is associated with a particular clinical feature of RA, such as positivity for RF. The samples presented here would be RF+ and RF– patients. Our four data classifications make it convenient for researchers to access and query RADB for a specific purpose.

To obtain all the studies from a certain population, and to compare data for the same polymorphism in different populations, we collected population information that includes detailed geographical information, and we provide a corresponding method of query. Currently, RADB contains data from 68 countries (see Supplementary File S2 on the Web site: http://www.bioapp.org/research/RA); however, only eight populations comprise >70% of the studies: Spain (96 studies), China (92 studies), UK (64 studies), Korea (55 studies), Japan (52 studies), USA (31 studies), Holland (31 studies), Sweden (24 studies), Poland (23 studies) and France (23 studies). Patients with RA are found worldwide, and the prevalence has been estimated at ∼1% (2). Interestingly, the prevalence is higher (>2%) in some Native American populations, and is lower (<0.3%) in East Asian, Southeast Asian and African populations (68). More research on different populations will be beneficial to the understanding of the different genetic mechanisms involved in RA.

Compared with analysis at the single-gene level, GO term enrichment analysis may provide further insight into the biological function of RA-related genes at the system level. GO term enrichment analysis for RA-related genes can be performed using Fisher’s exact test as implemented in the topGO package (69). A total of 477 genes (at least one polymorphism with a *P*-value < 0.05) have been associated with RA, and 364 of them have been successfully assigned GO terms. Table 2 lists the top 40 most significant GO terms (for more details, see Supplementary File S3 on the Web site: http://www.bioapp.org/research/RA), which include ‘inflammatory response’, ‘antigen processing and presentation’ and ‘cytokine imbalances’; this is in agreement with a previous study (70). Over the past half century, several hypotheses have been proposed to explain the pathogenesis of RA. The key hypotheses are (i) the immune complex hypothesis and (ii) the T cells and cytokines hypothesis. The immune complex hypothesis states that immune complexes formed by antibodies and anti-antibodies (RFs) activate the complement cascade, which releases chemotactic factors such as C5a, resulting in inflammation and tissue damage (71). The T cells and cytokines hypothesis suggests that an imbalance between T helper 1 and T helper 2 cells and changes in cytokine expression (e.g. IL1, TNF-α and IL6) cause the immunopathological damage observed in RA (72). There is a close relationship between the enriched GO terms and these hypotheses. ‘Inflammatory response’ (GO:0006955 and GO:0006954) is a prominent characteristic of RA; ‘antigen processing and presentation’ (GO:0019882) is the initial step in the immune response (73). Moreover, ‘cytokine imbalances’ (GO:0001817) have been shown to be associated with many immunological processes, including promoting autoimmunity, chronic inflammation and tissue damage. Our results of GO term enrichment analysis show that the pathogenesis of RA is very complex. We suggest that more attention should be given to the enriched GO terms and the genes annotated to these categories.

**Table 2.** Top 40 most significant GO terms associated with RA.

GOID	TERM	Annotated	Significant	Expected	*P*-value
GO:0006955	Immune response	690	111	17.79	4.69E-58
GO:0002376	Immune system process	998	130	25.73	5.26E-58
GO:0002682	Regulation of immune system process	385	73	9.93	5.48E-42
GO:0048583	Regulation of response to stimulus	465	75	11.99	3.85E-38
GO:0050776	Regulation of immune response	226	52	5.83	7.16E-34
GO:0002684	Positive regulation of immune system process	238	52	6.14	1.06E-32
GO:0050896	Response to stimulus	3502	194	90.30	6.22E-32
GO:0006952	Defense response	615	73	15.86	2.68E-28
GO:0050865	Regulation of cell activation	175	41	4.51	6.38E-27
GO:0051239	Regulation of multicellular organismal process	937	87	24.16	2.87E-26
GO:0002694	Regulation of leukocyte activation	166	39	4.28	1.21E-25
GO:0006954	Inflammatory response	325	50	8.38	3.72E-24
GO:0042221	Response to chemical stimulus	1281	99	33.03	5.39E-24
GO:0048584	Positive regulation of response to stimulus	236	43	6.09	1.13E-23
GO:0001817	Regulation of cytokine production	181	38	4.67	3.78E-23
GO:0051249	Regulation of lymphocyte activation	148	35	3.82	4.24E-23
GO:0031347	Regulation of defense response	143	34	3.69	1.62E-22
GO:0050863	Regulation of T cell activation	117	31	3.02	5.35E-22
GO:0050778	Positive regulation of immune response	145	33	3.74	3.13E-21
GO:0006950	Response to stress	1685	110	43.45	5.19E-21
GO:0048518	Positive regulation of biological process	2033	123	52.42	5.31E-21
GO:0051704	Multiorganism process	681	66	17.56	1.20E-20
GO:0002697	Regulation of immune effector process	101	28	2.60	1.97E-20
GO:0080134	Regulation of response to stress	274	42	7.07	3.28E-20
GO:0051240	Positive regulation of multicellular organismal process	244	39	6.29	2.06E-19
GO:0010033	Response to organic substance	721	66	18.59	2.39E-19
GO:0002237	Response to molecule of bacterial origin	86	25	2.22	8.96E-19
GO:0009605	Response to external stimulus	914	74	23.57	1.00E-18
GO:0009607	Response to biotic stimulus	384	47	9.90	1.38E-18
GO:0050867	Positive regulation of cell activation	111	27	2.86	3.73E-18
GO:0019882	Antigen processing and presentation	83	24	2.14	5.74E-18
GO:0009611	Response to wounding	530	54	13.67	8.65E-18
GO:0001775	Cell activation	287	40	7.40	8.77E-18
GO:0002696	Positive regulation of leukocyte activation	106	26	2.73	1.40E-17
GO:0002819	Regulation of adaptive immune response	56	20	1.44	6.63E-17
GO:0050670	Regulation of lymphocyte proliferation	83	23	2.14	8.69E-17
GO:0048519	Negative regulation of biological process	1812	106	46.72	1.02E-16
GO:0070663	Regulation of leukocyte proliferation	84	23	2.17	1.15E-16
GO:0032944	Regulation of mononuclear cell proliferation	84	23	2.17	1.15E-16
GO:0051251	Positive regulation of lymphocyte activation	97	24	2.50	2.50E-16

RADB is a genetic database that has been developed for basic research and clinical application for RA. RADB has several advantages over OMIM and GAD. First, more detailed phenotypic data are provided in RADB. Second, RADB contains the genotype data of RA-related polymorphisms, which are not given in the other genetic databases. Third, meta-analysis can be directly performed in RADB. Last but not least, RADB offers an easy user interface and the data can be easily compared.

In the future, we intend to add proteomic and epigenetic information to RADB, to reflect the growing importance of mRNA expression, DNA methylation and microRNAs in the pathogenesis of RA (74–76). Because of its ability to integrate and analyze the data from different sources, we believe that RADB will be helpful in studying and identifying the genetic and molecular basis of RA.

## Supplementary data

Supplementary data are available at Database Online.

## Funding

This work was supported in part by grants from the National Natural Science Foundation of China (31200934, 61300116, 81172842 and 81300945) and the Natural Science Foundation of Heilongjiang Province (grant numbers C201206 and QC2013C063). Funding for open access charge: 31200934, 81172842 and C201206.

*Conflict of interest*. None declared.
